# Associations of plasma biomarkers of Alzheimer’s disease pathology with modifiable risk factors and cognitive and functional outcomes: evidence from cross-sectional prediction and latent path analyses in the Bio-Hermes-001 cohort

**DOI:** 10.1186/s13195-026-02106-4

**Published:** 2026-06-18

**Authors:** Megan Kirk, Joseph Kwon, Kalliopi Mavromati, Adam Gordon-Boyle, Kamaldeep Bhui, Ivan Koychev, Sarah Bauermeister, Apostolos Tsiachristas

**Affiliations:** 1 Nuffield Department of Primary Care Health Sciences, University of Oxford, Radcliffe Primary Care Building, Radcliffe Observatory Quarter, Woodstock Road, Oxford, England OX2 6GG UK; 2 School of Cardiovascular and Metabolic Health, University of Glasgow, University Avenue, Glasgow, Scotland G12 8QQ UK; 3 Medical Sciences Division, University of Oxford, John Radcliffe Hospital, Headly Way, Headington, Oxford, England OX3 9DU UK; 4 Department of Psychiatry, Metabolic Psychiatry and Behavioural Medicine, University of Oxford, Warneford Hospital, Warneford Lane, Oxford, England OX3 7J3 UK; 5 Department of Brain Sciences, Imperial College London, Burlington Danes, The Hammersmith Hospital, Du Cane Road, London, England W12 ONN UK

**Keywords:** Dementia, Cognitive impairment, Functional decline, Neurodegeneration, Alzheimer’s Disease, Blood biomarkers, Depression, Body mass index, Latent path analysis

## Abstract

**Introduction:**

Plasma biomarkers of Alzheimer’s Disease and Related Dementias (ADRD) pathology may help clarify associations between modifiable risk factors (MRFs) and cognitive and functional impairment.

**Methods:**

Cross-sectional data from 1002 adults (mean age 72.0 ± 6.7) enrolled in the Bio-Hermes-001 study were analysed. First, block regression models assessed the added predictive value of MRFs *and* ADRD plasma biomarkers on cognitive (Mini-Mental State Examination, [MMSE]) and functional outcomes (Functional Activities Questionnaire, [FAQ]), beyond MRFs alone. Second, latent path analysis (LPA) examined potential pathways linking MRFs, ADRD biomarkers, and cognition and functional outcomes.

**Results:**

ADRD biomarkers significantly improved prediction models for MMSE (Δ*R*^*2*^ = 0.11) and FAQ scores (Δ*R*^*2*^ = 0.11). Indirect effects consistent with mediation were observed for depression (*B* = -0.05), BMI (*B* = 0.02), and tobacco use (*B* = 0.14) in relation to cognitive and functional outcomes.

**Discussion:**

ADRD biomarkers are relevant to pathways linking MRFs with cognitive and functional outcomes and have potential for risk stratification and prevention research. Longitudinal studies are needed to determine temporal relationships and clarify potential causal mechanisms.

**Supplementary Information:**

The online version contains supplementary material available at 10.1186/s13195-026-02106-4.

## Background

Dementia is one of the world’s most pressing health challenges. Disease cases are projected to surge from 55 million in 2019 to over 153 million by 2050 [1]. This places an unprecedented burden on already overstretched healthcare systems. Globally, dementia costs are estimated to exceed $9.2 trillion (USD) [2] within the next twenty years, becoming one of the costliest healthcare conditions. Unlike other leading causes of death, such as cardiovascular disease and cancer, dementia remains without an effective treatment or prevention strategy.

Once diagnosed, Alzheimer’s Disease (AD), accounting for 60–80% of dementia cases, and other related dementias (e.g., frontotemporal or vascular dementia) [3], are irreversible and progressive, causing debilitating memory loss and thinking problems, mood and behavioural symptoms, impaired function and quality of life, and ultimately death [3, 4]. Even before symptoms appear, a marked decline in brain glucose and lipid metabolism, especially in astrocytes and microglia cells, starts to occur [5, 6]. This metabolic disruption can start 10–20 years before clinical symptoms appear and often coincides with nerve cell loss, changes in brain structure (e.g., white matter atrophy) and blood vessels, cerebral insulin resistance and mitochondrial dysfunction, and oxidative stress [7–9]. Classic Alzheimer’s Disease and related dementias (ADRD) neuropathology includes buildup of beta-amyloid (Aβ) plaques and tau tangles in nerve cells, interfering with synaptic function and eventually leading to neuronal cell death [10, 11]. Neuroinflammation, a central mechanism driving disease progression, releases harmful inflammatory cytokines (e.g., interleukin-6 (IL-6), tumour necrosis factor-alpha (TNF-α), and C-reactive protein (CRP)) that further exacerbate disease progression by damaging synapses and compromising blood–brain barrier integrity [12–14]. Co-pathologies are typically present in later-stage dementias (e.g., vascular disease, Lewy bodies, and TAR DNA-binding protein 43 (TDP-43) accumulation) and rapidly accelerate the disease [15, 16].

While there is no cure for dementia, novel amyloid clearance therapies (e.g., Lecanemab) for ADRD are being introduced imminently, but these monoclonal antibody therapies act on a single upstream target (Aβ). Systematic reviews of phase III monoclonal antibody clinical trials show only modest cognitive benefits, and present concerns around contradictory results, long-term efficacy, safety concerns, and inhibitory costs [17, 18]. These challenges highlight the need for complementary approaches that identify earlier, modifiable pathways contributing to cognitive decline. Understanding blood-based biomarkers of neurodegenerative pathology offers a less expensive, non-invasive way to examine how lifestyle and other MRFs may influence cognitive and functional outcomes.

According to the 2024 *Lancet Commission* on dementia prevention, nearly half of dementia cases (45%) are largely attributable to 14 preventable MRFs [19]. Newly added to the list is high low-density lipoprotein (LDL) cholesterol which supports a growing interest in addressing the metabolic features of AD. Indeed, a growing body of research now links dementia with depression and cardiometabolic risk factors, such as obesity, type 2 diabetes mellitus, hypertension, and hypercholesterolemia [20–22]. Shared across these conditions is systemic inflammation, seen in raised plasma levels of IL-6, TNF-α, and CRP [23–25]. Persistent inflammation weakens the blood–brain barrier, worsening nerve cell damage and cognitive impairment [26]. Late-life depression is associated with a two- to fivefold increase in dementia risk via cerebrovascular changes, hormonal and neuroendocrine dysfunction including hypothalamic–pituitary–adrenal (HPA) axis dysregulation and elevated cortisol levels linked with metabolic syndromes, and hippocampal atrophy involved in memory loss [27, 28]. Depression alters glucocorticoid steroid levels and worsens insulin resistance, increases mood instability, reinforcing the links between depression, metabolism, and brain health [21, 27, 28].

Lifestyle behaviours like smoking and excessive alcohol further contribute to metabolic changes via inflammation and oxidative stress [29, 30], harming mental health [23, 31], cardiometabolic health [32], and cognitive function [20, 24, 33]. Despite robust evidence on individual MRFs, the 2024 *Lancet Commission* suggests examining combinations of MRFs and underlying biological mechanisms influencing cognitive and functional impairment to inform more precise prevention approaches [19]. Specifically, exploring the combined influence of depression, cardiometabolic health, lifestyle factors, and dementia, especially through inflammatory pathways and plasma ADRD biomarkers of neurodegenerative pathology, may lead to earlier, more cost-effective strategies to slow or stop dementia [22, 34].

Advances in blood-based biomarker assessment have enabled minimally invasive, early detection of ADRD-related neuropathology before the onset of clinical symptoms, and include plasma Aβ, phosphorylated tau pathology (pTau), and markers of neurodegenerative pathology (e.g., neurofilament light [NfL]) and glial activation (e.g., glial fibrillary acidic protein [GFAP]) [35]. Aβ and pTau show a high specificity for ADRD-related pathology and have shown to be robust predictors of longitudinal cognitive decline [36]. Plasma NfL is a validated marker of neuroaxonal injury and risk of future disease progression, but is non-specific to AD and can be present in other conditions [37]. GFAP, a marker of astrocytic activation related to amyloid pathology [38], has shown to increase in the preclinical phase of dementia and predict incident dementia and cognitive decline in large cohort studies (e.g., UK Biobank [37]); however, it is also non-specific to AD and may also be elevated in other neurological and systemic conditions. Together, these plasma biomarkers reflect an important biological domain relevant to ADRD pathology and may also reflect the biological impact of MRFs across the life course.

Prior studies have reported associations between vascular, metabolic, and lifestyle risk factors and plasma measures of Aβ, phosphorylated tau (pTau), and neurodegeneration, suggesting that MRF exposures may influence cognitive outcomes through biomarker-detectable neuropathological processes [39]. Longitudinal cohort studies demonstrate that baseline plasma biomarkers, particularly pTau-217, are detectable in cognitively unimpaired, Aβ positive individuals and predict cognitive decline and ADRD disease progression [40]. In the German AgeCoDe cohort, higher baseline plasma levels of pTau-181, NfL and GFAP were significantly associated with faster cognitive decline and an increased risk of progression to AD over an 8-year follow-up [41]. Additional evidence links cardiometabolic and lifestyle factors to variations in plasma ADRD biomarkers [42, 43], including associations between these biomarkers and cognitive performance in cognitively healthy and clinically impaired samples. For example, healthier lifestyle behaviours (e.g., physical activity and nutrition) have been associated with more favourable plasma ADRD biomarker profiles [43], and chronic vascular (e.g., hypertension) and metabolic conditions (e.g., Type 2 Diabetes Mellitus) are associated with higher plasma biomarker pathology and cognitive impairment risk [42, 43]. However, most prior studies have examined these relationships in isolation, without jointly evaluating the predictive utility of plasma biomarkers and assessing their potential role linking MRFs to cognitive and functional outcomes. This present study addresses this gap by integrating prediction and latent path analyses within a single analytic framework to provide insight into the clinical relevance of these biomarkers and their potential involvement in relationships between key MRFs and cognitive and functional impairment in late-life. We first explored whether the inclusion of ADRD biomarkers improved prediction models of cognition and functional impairment. We then examined whether these biomarkers were involved in pathways linking MRFs and cognitive and functional outcomes to better characterise potential relationships among these variables. We hypothesised that: 1) MRFs would be associated with poorer cognition and functional outcomes, 2) the inclusion of ADRD plasma biomarkers of neurodegeneration would improve outcome prediction, and 3) that these biomarkers would show indirect associations between MRFs and cognition and functional impairment.

## Methods

### Study design and cohort

We performed cross-sectional analyses of 1002 older adults, aged 60–85 years, enrolled in the Bio-Hermes-001 cohort study previously conducted from April 2021 to November 2022 across 17 research sites in the USA [44]. Cognitive classification of participants into Cognitively Normal (CN; *n* = 417), Mild Cognitive Impairment (MCI; *n* = 313), and probable/mild AD (PAD; *n* = 272) groups was determined previously based on site-administered clinical assessments that followed standardised diagnostic criteria and data collection methods detailed in a prior publication from the Bio-Hermes-001 study [44].

### Health outcomes

#### Cognitive and functional health outcomes

*Global cognitive status* was assessed using the Mini-Mental State Examination (MMSE), a widely validated 30-point brief clinician-administered screening tool developed to rapidly assess cognitive status [45, 46]. Scores range from 0–30 with higher scores reflecting better global cognitive performance. A threshold score of < 23 is suggestive of cognitive impairment. Eligible participants in the Bio-Hermes-001 sample had an MMSE score ranging from 20 to 30. In three cases, MMSE scores < 20 were included based on clinical judgement. CN MMSE scores ranged from 26–30; MCI MMSE scores ranged from 24–30; PAD MMSE scores ranged from 17–24 [44]. Total score was used as the global cognitive outcome in our analyses.

*Functional impairment* was assessed using the Functional Activities Questionnaire (FAQ) [47]. The FAQ is a 10-item questionnaire with scores ranging from 0–30, and higher scores indicating greater functional impairment. A cut-point of 9 (e.g., dependent in 3 + activities) indicates impaired function and possible cognitive impairment [47].

### Predictors

#### Sociodemographic factors

Self-reported sociodemographic variables of age (continuous), sex (dichotomous), ethnicity (categorical), and education (continuous (total number of years of education)) and dichotomous (post-secondary education, ≥ 13 yrs vs. secondary education < 13 years of education) were examined in the initial model to statistically control for baseline differences and reduce potential confounding, ensuring accurate estimation of the independent effects of MRFs.

#### Modifiable risk factors of dementia

The selection of MRFs was guided by the 2024 *Lancet Commission*, the available data in the cohort dataset, and established associations with cognitive performance, functional impairment, and plasma biomarkers of ADRD pathology, including their potential for modification through clinical or public health interventions. The following MRFs were included:

1. *Depressive symptoms* were measured using the 15-item short-form Geriatric Depression Scale (GDS) [48] with scores ranging from 0–15. Scores ≥ 5 indicates the presence of elevated depressive scores. GDS continuous scores (0–15) and a binary variable (high depression scores, ≥ 5 vs. low depression scores, < 5) were used in analyses;
2. *Low-density lipoprotein (LDL) Cholesterol* obtained via plasma lipid panel were included as a continuous variable or dichotomous variable (high LDL, ≥ 3.4 mmol/L vs. low LDL < 3.4 mmol/L) to indicate threshold elevated LDL based on established clinical threshold criteria [49];
3. *Blood Pressure (BP)* was analysed as a continuous variable using systolic blood pressure (SBP) and dichotomised to reflect the presence or absence of hypertension based on NICE clinical guidelines (high BP, diastolic blood pressure ≥ 90 mmHg or SBP ≥ 140 mmHg vs. normal BP) [50];
4. *Body Mass Index (BMI)* was analysed as a continuous variable and a binary variable indicating the presence or absence of obesity (obese, BMI ≥ 30 vs. non-obese) based on NIH clinical classification [51];
5. *Alcohol and tobacco use* were both assessed via a single-item self-report question of alcohol or tobacco use with a binary ‘yes/no’ response to either current use, past use, or never. For both variables, a binary (yes/no) indicator was created to reflect presence versus absence of exposure (current/past exposure vs. no exposure). Information on frequency, quantity, duration, or recency of alcohol or tobacco use was not assessed in the original Bio-Hermes-001 dataset.

#### Blood biomarkers of neurodegenerative pathology

Blood plasma samples of the Bio-Hermes-001 cohort were previously collected and included the following ADRD pathology biomarkers: plasma beta-amyloid (Aβ) Aβ40, Aβ42, Aβ42/40 ratio, total plasma tau (tTau), phosphorylated tau (pTau181, pTau217), glial fibrillary acidic protein (GFAP), plasma neurofilament light (NfL), and genetic biomarker of apolipoprotein E ε4 (APOE ε4) carrier status [44]. We note that GFAP is a sensitive marker of astrocyte reactivity and may reflect early amyloid-related changes; however, GFAP is non-specific to ADRD pathology and can occur in other conditions. Plasma biomarker analyses were conducted at different laboratories: Aβ40 and Aβ42 from C_2_N; pTau181, GFAP, NfL and APOE ε4 from Roche; and pTau217 from Eli Lilly and analytic protocols for all biological data have been previously described in the original Bio-Hermes-001 cohort publication [44]. Previous analysis found no statistically significant difference in plasma tTau level between amyloid positive and negative subgroups, and hence tTau was not used in our study as a predictive biomarker [44]. Of the Bio-Hermes-001 cohort, 956 (95.4%) (n = 400 for CN, n = 297 for MCI, n = 259 for PAD) completed positron emission tomography (PET) or cerebrospinal fluid (CSF) measurement of Aβ level (98.8% by PET). Aβ positivity (AP) was defined using a previously standardised uptake ratio (SUVR) cutoff of 1.1, which corresponds to a Centiloid scale value of 24.1 from PET/CSF Aβ assessments previously used by the Bio-Hermes study team and incorporated into the curated dataset for analysis [44, 52, 53].

### Data cleaning and processing

The methods for processing the variables, including imputations of blood biomarker values and removal of outliers, are detailed in Table S1 in the Supplementary File 1. In brief, imputations were made to pTau181 and pTau217 values below the lower or above the upper limits of quantitation according to the steps used previously [44]. Blood biomarker values that exceeded six standard deviations (SDs) from the sample mean were treated as statistical outliers and excluded to mitigate undue influence on regression coefficients. The Aβ42/40 ratio (range 0.07–0.15) and pTau217 (range 0.001–1.39) values were multiplied by 100 to ease interpretation of their coefficients in multivariate regressions.

### Statistical analysis

All analyses were conducted using RStudio (version 4.1.2) available on AD Workbench hosted by AD Data Initiative [54]. Complete individual datasets, except for the imputations of pTau181 and pTau217 described above, were used for all analyses.

Descriptive statistics, including means and standard deviations for continuous variables and frequencies and percentages for categorical variables were reported by cognitive status (i.e., Cognitively normal, MCI, and PAD) and differences were statistically tested using analysis of variance (ANOVA), and chi-square tests. Exploratory bivariate associations between MRFs and plasma ADRD biomarkers and MMSE and FAQ scores were initially assessed prior to regression analyses to help conceptualise the model (Table S2 Supplementary File 1). A hierarchical multiple regression method using conceptually driven blocks of predictors was performed to systematically evaluate incremental variance explained by MRFs, allowing clear interpretation of the separate contributions and incremental predictive value of variables cumulatively from sociodemographic variables, lifestyle factors, depressive symptoms, cardiovascular risk, and plasma ADRD neuropathology in relation to cognitive and functional outcomes [55, 56]. The following blocks of variables were entered cumulatively:

- Model 1: (Baseline Adjustment Set) Sociodemographic factors
- Model 2: Addition of Lifestyle Factors (BMI, alcohol use, tobacco use);
- Model 3: Addition of Depressive Symptoms (GDS);
- Model 4: Addition of Cardiometabolic Risk Factors (LDL, SBP);
- Model 5: Addition of Plasma ADRD Biomarkers of Neuropathology (Aβ42/40, pTau181, pTau217, GFAP, NfL, APOE ε4);
- Model 6: Addition of Functional impairment (FAQ) or Cognitive Performance (MMSE).

The hierarchical order of variable inclusion was guided by a theory-driven, life course conceptual framework of dementia rather than data-driven considerations, consistent with recommendations for blockwise regression modelling in life-course epidemiological cognitive aging research [57]. Variables were entered according to their degree of modifiability, temporal precedence, and presumed causal proximity to cognitive and functional outcomes [57], as well as guided by the 2020 and 2024 *Lancet Commission on dementia prevention, intervention, and care* where possible [19, 58]*.* This approach allowed for evaluation of incremental predictive contribution of each domain while controlling for all other variables entered in previous steps. Regression analyses were performed using the ‘lm()’ function included in the standard statistical package in RStudio to estimate the association between MMSE or FAQ as the dependent variable with ADRD plasma biomarkers of neurodegeneration and MRFs as predictors. In Model 1, Self-reported sociodemographic factors for age (continuous), sex (male vs. female), years of education (continuous) and race/ethnicity (non-Hispanic White vs. other) were entered first to statistically control for non-modifiable background factors that may potentially confound both cognitive outcomes and ADRD biomarkers. These variables reflect early-life, fixed characteristics that precede all subsequent exposures and formed the baseline adjustment set to assess incremental contributions independent of demographic factors. In Model 2 (lifestyle factors), BMI, alcohol use, and tobacco use were introduced as the first modifiable block reflecting behaviourally modifiable determinants that emerge in mid-life and may influence mental health, cardiometabolic health, and neurobiological processes [19]. The *Lancet Commission* identifies lifestyle behaviours as foundational intervention targets that contribute to cumulative risk of ADRD through multiple biological and psychosocial pathways [19]. In Model 3, depressive symptoms (GDS) were added as the next block based on depression conceptualised as an intermediate factor that may link lifestyle behaviours to cardiometabolic dysfunction and neurodegeneration. This allowed us to evaluate the incremental explanatory value of depression beyond behavioural risk factors, while minimising overadjustment. In Model 4, cardiometabolic risk factors (LDL, SBP) were entered as a subsequent block since these factors are more proximal to neurodegeneration and are established as key mechanistic links between lifestyle and mental health exposures and later cognitive decline. Their placement reflects progression from upstream behaviours to downstream physiological dysregulation. In Model 5, plasma ADRD neurodegenerative biomarkers were introduced to assess whether molecular indicators of neurodegeneration could further explain variance in cognitive or functional outcomes above and beyond established modifiable risk factors. In Tables S3 and S4 in Supplementary File 1, we first explored each ADRD plasma biomarker as an independent predictor prior to combining them in Model 5 ensuring robustness with respect to model specification. We assessed multicollinearity using variance inflation factor (VIF) values and no values exceeded > 3.0 indicating no collinearity issues. We elected to report unstandardised beta coefficients and p-values to preserve interpretability of effect sizes in the original measurement units of the clinical outcomes and predictors, and to allow direct interpretation of the expected change in outcome associated with a one-unit change in the predictor. Finally, in Model 6 we incorporated either cognitive performance (MMSE) or functional impairment (FAQ) as the primary outcome, with the other entered as a subsequent predictor to evaluate the incremental contribution of all preceding risk domains to clinical outcomes of interest, while acknowledging the bidirectional relationship between cognition and daily functioning. Model fit indices included adjusted *R*^*2*^ values, *F-*statistic, and Akaike Information Criterion (AIC) and Bayesian Information Criterion (BIC) values. AIC and BIC values were divided by the sample size of each model since the values vary proportionately with sample size. Bonferroni correction was applied when interpreting the models to account for increased risk of type I error (false positives) when conducting multiple tests of significant association [59]. An adjusted significance threshold was derived by dividing the original threshold α = 0.05 by the number of covariates (excluding the intercept) in the given regression model.

**Fig. 1:**
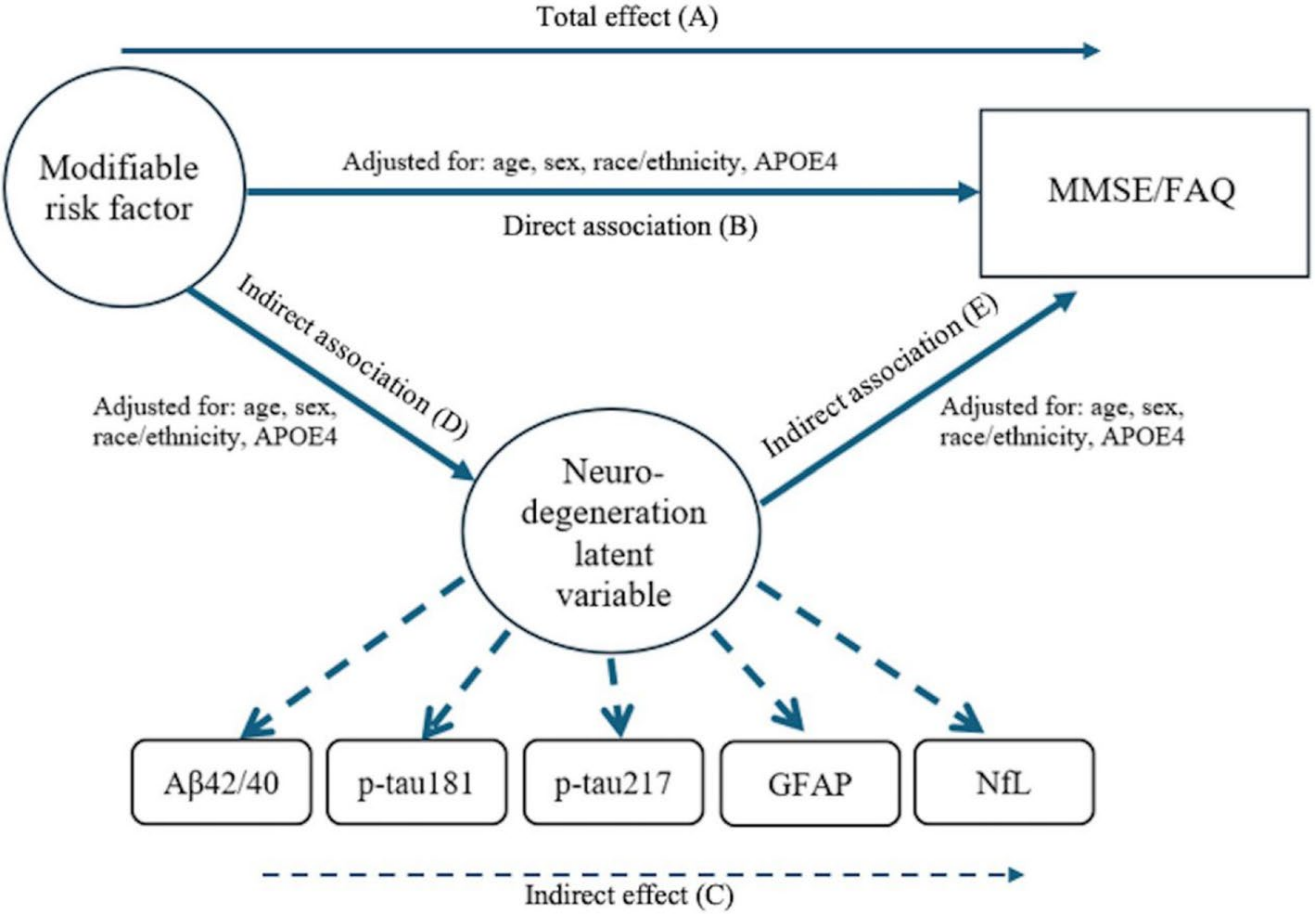
Directed acyclic graph for first and second sets of latent path analyses. *Abbreviation:* Aβ: beta-amyloid; APOE ε4: apolipoprotein E ε4; GDS: Geriatric Depression Scale; GFAP: glial fibrillary acidic protein; MMSE: Mini-Mental State Examination; NfL: neurofilament light; pTau: phosphorylated tau

Subsequently, a series of cross-sectional latent path analyses (LPA) [60] were performed using the ‘lavaan’ package [61] in RStudio to examine the direct and indirect pathways linking MRFs to cognitive (MMSE) and functional (FAQ) outcomes. A latent construct representing plasma ADRD neurodegenerative disease pathology was specified using five plasma ADRD biomarkers (Aβ42/40 (reverse scored), pTau181, pTau217, GFAP, and NfL) to assess indirect effects of underlying ADRD neuropathology. Separate LPAs were performed for each MRF. The second set of LPAs replaced MMSE with FAQ as the outcome of interest. We present the directed acyclic graph for the structure of the LPAs in Fig. 1. The point estimates and standard errors of the direct, indirect, and total effects were estimated. The total effect (A) equals the sum of the direct association (B) and the sum (C) of the indirect associations through ND (D + E) so that: A = B + C, with C = (D + E).

The strength of correlation between the observed blood biomarkers and the latent ADRD neurodegenerative disease pathology construct was assessed through factor loadings, with a loading of ≥ 0.4 judged to be adequate for each observed variable [62]. When assessing the empirical fit of LPAs, the root mean square error of approximation (RMSEA) and the standardised root mean square residual (SRMR) of less than 0.05 indicated good fit, whilst values between 0.05 and 0.08 indicated reasonable fit [62]. The comparative fit index (CFI) [63] and the Tucker and Lewis Index (TLI) [64] values above 0.95 indicated good fit, whilst values between 0.90 and 0.95 indicated reasonable fit.

## Results

### Participant characteristics and demographics

The baseline sociodemographic and clinical characteristics of the 1002 participants analysed are presented in Table 1. Significant age differences were observed across groups, with CN participants being younger (*M* = 70.3, SD = 6.4) compared to those with MCI (*M* = 72.2, SD = 6.8), or PAD (*M* = 74.4, SD = 6.1; *p* < 0.001). The CN group had a higher proportion of females (61.2%) compared to MCI (53.7%) and PAD (51.5%; *p* = 0.024). Years of education also significantly declined with increasing cognitive impairment (*p* < 0.001).

**Table 1:** Sample characteristics and demographics stratified by cognitive health status (*n* = 1002)

	**Cognitively normal [*n* = 417]**	**Mild cognitive impairment [*n* = 313]**	**Probable Alzheimer’s Disease [*n* = 272]**	***P*-value**	**Total population [*n* = 1,002]**
Age (years), mean (SD)	70.3 (6.4)	72.2 (6.8)	74.4 (6.1)	<.001	72.0 (6.7)
Male/female, n/n, (% female)	162/255 (61.2)	145/168 (53.7)	132/140 (51.5)	.024	439/563 (56.2)
Non-Hispanic White, n, %	337 (80.8)	236 (75.4)	182 (66.9)	<.001	755 (75.3)
Years of education, mean (SD)	15.8 (2.5)	15.3 (2.8)	14.7 (3.1)	<.001	15.3 (2.8)
Post-secondary Education (≥ 13 years), n, %	217 (88.2)	155 (79.5)	123 (71.1)	<.001	495 (80.6)
LDL^a^, high/low, n/n (% high)	57/325 (14.9)	37/245 (13.1)	27/170 (13.7)	.794	121/740 (14.1)
Triglycerides^b^ high/low n/n (% high)	74/311 (19.2)	45/239 (15.8)	32/165 (16.2)	.462	151/715 (17.4)
Total cholesterol^c^ high/low^d^, n/n (% high)	82/296 (21.7)	53/225 (19.1)	32/159 (16.8)	.356	167/681 (19.7)
Blood pressure, high/low^e^ n/n (% high)	138/279 (33.1)	105/208 (33.5)	119/153 (43.8)	.009	362/640 (36.1)
Obese^f^ Yes/No, n/n, (%yes)	150/267 (36.0)	80/233 (25.6)	67/203 (24.8)	.001	297/703 (29.7)
Current or past use of alcohol, n (% yes)	313 (75.1%)	224 (71.6)	171 (62.9)	.003	708 (70.7)
Current or past use of tobacco n, % yes	155 (37.2)	134 (42.8)	98 (36.0)	.177	387 (38.6)
GDS depression high/low^g^ n/n, (% yes)	25/392 (6.0)	45/267 (14.4)	34/238 (12.5)	<.001	104/897 (10.4)
APOE ε4 carrier n, %	137 (33.1)	112 (36.7)	118 (45.2)	.006	367 (37.4)
Amyloid positive in PET or CSF Yes/No, n/n (% yes)	85/313 (21.4)	104/190 (35.4)	156/104 (60.0)	<.001	345/607 (36.2)
MMSE total, mean (SD)	28.4 (1.5)	27.1 (2.0)	23.2 (2.5)	<.001	26.6 (2.9)
FAQ total, mean (SD)	0.8 (1.6)	3.6 (4.5)	9.5 (6.9)	<.001	4.0 (5.7)

*Abbreviation*: *AD* Alzheimer’s disease, *APOE ε4* apolipoprotein E allele 4, *BMI* body mass index, *CSF* cerebrospinal fluid, *DBP* diastolic blood pressure, *FAQ* Functional Activities Questionnaire, *GDS* Geriatric Depression Scale, *HDL* high-density lipoprotein, *LDL* low-density lipoprotein, *MMSE* Mini-Mental State Examination, *PET* positron emission tomography, *SBP* systolic blood pressure, *SD* standard deviation

^a^ High if LDL ≥ 3.4 mmol/L

^b^ High if triglyceride ≥ 2.3 mmol/L

^c^ Estimated as: LDL + HDL + 0.2*Triglyceride

^d^ High if total cholesterol ≥ 5.2 mmol/L

^e^ High if DBP ≥ 90 mmHg or SBP ≥ 140 mmHg

^f^ Obese if BMI ≥ 30

^g^Depressed if GDS ≥ 5

Elevated blood pressure was more common in PAD (43.8%) than in the CN (33.1%) and MCI (33.5%) groups (*p* = 0.009). BMI was significantly lower in PAD (27.0 vs. 28.4 in cognitively normal; *p* = 0.010), with a decreasing trend in overweight/obesity with increasing cognitive impairment (*p* = 0.009). Alcohol use was more frequent in the CN group (75.1%) than those with PAD (62.9%; *p* = 0.003). In terms of depressive symptoms (GDS ≥ 5), mean GDS scores were highest in MCI (*M* = 2.2, SD = 1.9) and PAD groups (*M* = 2.2, SD = 1.9) compared to the CN group (*M* = 1.5, SD = 1.6, *p* < 0.001). Global cognitive performance (MMSE scores) decreased from 28.4 (SD = 1.5) in CN adults to 23.2 (SD = 2.5, *p* < 0.001) in PAD, while FAQ scores increased from 0.3 to 9.5 (*p* < 0.001) across groups, reflecting greater functional impairment with worsening cognitive status.

### Regression models predicting global cognitive function (MMSE) and Functional Impairment (FAQ)

#### Global cognitive function (MMSE)

Regression models examining the incremental contribution of demographic, lifestyle, mental health, cardiometabolic and plasma ADRD biomarker predictors of global cognitive function (MMSE) are presented in Table 2. In Model 1, demographic variables (e.g., age, sex, education, and race/ethnicity) explained 15% of the variance in MMSE Scores (*Adj. R*^*2*^ = 0.15, *F* (4, 609) = 28.15, *p* < 0.0001). Younger age (*B* = −0.10, *p* < 0.001), non-Hispanic White ethnicity (*B* = 1.73, *p* < 0.001), and years of education (*B* = 0.21, *p* < 0.001) were significantly associated with better cognitive performance even after Bonferroni correction for multiple comparisons (adjusted significance threshold of *p* = 0.0125).

**Table 2:** Hierarchical regression analysis predicting global cognitive function (MMSE) score

Predictor	Model 1	Model 2	Model 3	Model 4	Model 5	Model 6
*Unstandardised Beta Coefficient (B*) [Standard Error]						
Intercept	29.45^***^ [1.36]	27.61^***^ [1.55]	28.14^***^ [1.56]	30.11^***^ [1.94]	32.21^***^ [2.53]	31.69^***^ [2.36]
Demographics						
Age	−0.10^***^ [0.02]	−0.10^***^ [0.02]	−0.10^***^ [0.02]	−0.10^***^ [0.02]	−0.07^***^ [0.02]	−0.06^**^ [0.02]
Sex (Female)	0.35 [0.22]	0.45^*^ [0.22]	0.42^*^ [0.22]	0.40 [0.25]	0.51^*^ [0.25]	0.23 [0.24]
Non-Hispanic White	1.73^***^ [0.25]	1.63^***^ [0.25]	1.66^***^ [0.25]	1.28^***^ [0.29]	1.50^***^ [0.28]	1.30^***^ [0.27]
Education	0.21^***^ [0.04]	0.21^***^ [0.04]	0.20^***^ [0.04]	0.18^***^ [0.05]	0.16^***^ [0.04]	0.14^***^ [0.04]
Lifestyle Factors						
BMI		0.03 [0.02]	0.02 [0.02]	0.03 [0.02]	0.01 [0.02]	0.00 [0.02]
Alcohol Use		0.81^***^ [0.24]	0.74^**^ [0.25]	0.88^**^ [0.27]	0.74^**^ [0.26]	0.51^*^ [0.24]
Tobacco Use		0.35 [0.23]	0.36 [0.22]	0.30 [0.24]	0.14 [0.23]	0.04 [0.22]
Mental Health						
Depression (GDS)			−0.13^*^ [0.06]	−0.15^*^ [0.07]	−0.10 [0.06]	−0.03 [0.06]
Cardiovascular Health						
LDL				−0.19 [0.14]	−0.27^*^ [0.14]	−0.22 [0.13]
SBP				−0.01 [0.01]	−0.00 [0.01]	−0.00 [0.01]
ADRD Biomarkers of Neurodegenerative Disease Pathology						
Aβ42/40					−0.24^*^ [0.14]	−0.22 [0.13]
pTau181					−0.37 [0.35]	−0.21 [0.33]
pTau217					−0.03^**^ [0.01]	−0.02^*^ [0.01]
NfL					−0.16^*^ [0.06]	−0.07 [0.06]
GFAP					0.00 [0.00]	0.00 [0.00]
Amyloid Positivity in PET/CSF					−1.18^***^ [0.32]	−0.86^**^ [0.30]
APOE ε4					0.43 [0.26]	0.45 [0.24]
Functional Impairment						
FAQ						−0.18^***^ [0.02]
*Model Fit Indices*						
*Adj. R*^*2*^	0.15	0.17	0.17	0.15	0.26	0.35
Δ*R*^*2*^		0.02	0.00	-.02	0.11	0.09
*F*	28.15^***^	18.80^***^	17.12^***^	10.15^***^	10.90^***^	15.82^***^
*n*	614	612	611	611	486	486
*AIC/n*	4.82	4.80	4.80	4.80	4.66	4.52
*BIC/n*	4.86	4.87	4.87	4.87	4.82	4.69

*AD* Alzheimer’s disease, *AIC* Akaike Information Criterion, *BMI* Body Mass Index, *CSF* cerebrospinal fluid, *FAQ* Functional Activities Questionnaire, *GDS* Geriatric Depression Scale, *GFAP* glial fibrillary acidic protein, *LDL* low-density lipoprotein, *MMSE* Mini-Mental State Examination, *NfL* neurofilament light, *PET* positron emission tomography, *SBP* Systolic Blood Pressure

^***^ *p* <.001

^**^ p <.01

^*^p <.05

The addition of lifestyle factors in Model 2 slightly improved model fit (*Adj. R*^*2*^ = 0.17, *F*(7, 604) = 18.80, *p* < 0.001). Alcohol use emerged as a positive predictor of MMSE scores (*B* = 0.81, *p* < 0.001). Depressive symptoms were added in Model 3. An inverse association between depressive symptoms with MMSE (*B* = – 0.13, *p* = 0.029) was found. However, the added explained variance was negligible (*Adj. R*^*2*^ = 0.17, *F*(8, 602) = 17.12, *p* < 0.001), and the association was not significant under Bonferroni correction (adjusted threshold *p* = 0.0063). In Model 4, the addition of cardiometabolic variables did not improve the model (Δ*R*^*2*^ =—0.02), and neither variable significantly predicted cognitive scores. However, in Model 5, plasma biomarkers of ADRD pathology were added collectively and yielded a substantial increase in explained variance by 11 percentage points compared to Model 4 (*Adj. R*^*2*^ = 0.26, Δ*R*^*2*^ = 0.11, *F*(17, 468) = 10.90, *p* < 0.001*).* Amyloid positivity in PET/CSF (*B* = −1.18, *p* < 0.001), higher NfL (*B* = −0.16, *p* = 0.013), and elevated pTau217 (*B* = −0.03, *p* = 0.005) were all significant predictors of lower MMSE scores, though the association was significant only for amyloid positivity in PET/CSF under Bonferroni correction (adjusted threshold *p* = 0.0029). Separate addition of each plasma biomarker, except for Aβ42/40, generated associations with MMSE that remained significant under Bonferroni correction (Supplementary File 1: Models 5A-5G in Table S3). In the final model (Model 6), adding functional impairment (FAQ) resulted in the greatest improvements in model fit (*Adj. R*^*2*^ = 0.35, *F*(18, 467) = 15.82, *p* < 0.001)*.* Higher FAQ scores predicted significantly lower MMSE scores (*B* = −0.18, *p* < 0.001). White ethnicity (*B* = 1.30, *p* < 0.001), higher level of education (*B* = 0.14, *p* < 0.001), and FAQ remained as significant predictors of MMSE after Bonferroni correction (adjusted threshold *p* = 0.0028). Overall, the final model explained 35% of the variance in MMSE score, with three sociodemographic factors and FAQ emerging as significant predictors even after adjusting for multiple comparisons.

#### Functional impairment (FAQ)

The incremental predictive value of MRFs and ADRD biomarkers on functional impairment are presented Table 3. Demographic factors accounted for 5% of the variance in FAQ (*Adj. R*^*2*^ = 0.05, *F*(4, 609) = 9.04, *p* < 0.001) in Model 1. In Model 2, the addition of lifestyle factors marginally improved model fit (*Adj. R*^*2*^ = 0.07, *F*(7, 604) = 7.34, *p* < 0.001). Alcohol use (*B* = −1.52, *p* = *0.0*03) was associated with lower functional impairment even under Bonferroni correction (adjusted threshold *p* = 0.0071). In Model 3, adding depressive symptoms (GDS) improved the model predicting FAQ by four percentage points (Δ*R*^*2*^ = 0.04) with higher GDS scores predicting greater functional impairment (*B* = 0.66, *p* < 0.001). As seen in Model 4, the addition of cardiometabolic risk factors had no added explanatory value. In Model 5, the addition of ADRD plasma biomarkers significantly improved model fit (*Adj. R*^*2*^ = 0.21, Δ*R*^*2*^ = 0.11, *F*(17, 468) = 8.70, *p* < 0.001) and explained a further 11% of variance in FAQ. Higher NfL (*B* = 0.48, *p* < 0.001) emerged as a significant predictor of greater functional impairment even under Bonferroni correction (adjusted threshold *p* = 0.0029). In Model 6, cognitive performance (MMSE) significantly improved model fit and accounted for an additional 11% of explained variance. Lower MMSE scores were associated with greater functional impairment (*B* = −0.73, *p* < 0.001). Four predictors emerged in the final model accounting for 32% of the variance in functional impairment (*Adj. R*^*2*^ = 0.32, *F*(18, 467) = 13.42, *p* < 0.001). Female sex (*B* = −1.16, *p* = 0.014), greater depressive symptoms (*B* = 0.32, *p* = 0.008), elevated NfL (*B* = 0.37, *p* < 0.001), and lower MMSE scores (*B* = −0.73, *p* < 0.001) predicted higher FAQ scores, with the associations for elevated NfL and lower MMSE scores remaining significant after Bonferroni correction (adjusted threshold *p* = 0.0028).

**Table 3:** Hierarchical regression analysis predicting functional impairment (FAQ) score

Predictor	Model 1	Model 2	Model 3	Model 4	Model 5	Model 6
*Unstandardised Beta Coefficients (B*) [Standard Error]						
Intercept	−2.92 [2.83]	1.44 [3.21]	−0.84 [3.16]	−5.16 [3.89]	−2.81 [5.05]	20.64^***^ [5.47]
Demographics						
Age	0.15^***^ [0.03]	0.13^***^ [0.03]	0.13^***^ [0.03]	0.13^***^ [0.04]	0.03 [0.04]	−0.01 [0.04]
Sex (Female)	−1.61^***^ [0.47]	−1.87^***^ [0.46]	−1.69^***^ [0.45]	−1.32^**^ [0.50]	−1.53^***^ [0.50]	−1.16^*^ [0.47]
Non-Hispanic White	−0.76 [0.53]	−0.53 [0.53]	−0.73 [0.52]	−0.34 [0.58]	−1.11^*^ [0.57]	−0.02 [0.54]
Education	−0.15 [0.08]	−0.15 [0.08]	−0.11 [0.08]	−0.10 [0.09]	−0.10 [0.09]	0.02 [0.08]
Lifestyle Factors						
BMI		−0.06 [0.04]	−0.06 [0.04]	−0.06 [0.04]	−0.01 [0.04]	−0.01 [0.03]
Alcohol Use		−1.52^**^ [0.50]	−1.08^**^ [0.50]	−1.44^**^ [0.54]	−1.26^*^ [0.52]	−0.72 [0.49]
Tobacco Use		−0.56 [0.47]	−0.58 [0.45]	−0.73 [0.49]	−0.55 [0.47]	−0.45 [0.44]
Mental Health						
Depression (GDS)			0.66^***^ [0.12]	0.54^***^ [0.13]	0.39^**^ [0.13]	0.32^**^ [0.12]
Cardiovascular Health						
LDL				0.28 [0.28]	0.31 [0.27]	0.11 [0.25]
SBP				0.03 [0.01]	0.01 [0.01]	0.01 [0.01]
ADRD Biomarkers of Neurodegenerative Disease Pathology						
Aβ42/40					0.16 [0.29]	−0.02 [0.27]
pTau181					0.89 [0.71]	0.62 [0.66]
pTau217					0.02 [0.02]	0.01 [0.02]
NfL					0.48^***^ [0.13]	0.37^**^ [0.12]
GFAP					0.00 [0.01]	0.00 [0.00]
Amyloid Positivity in PET/CSF					1.75^**^ [0.64]	0.90 [0.61]
APOE ε4					0.10 [0.52]	0.41 [0.49]
Cognitive Function						
MMSE						−0.73^***^ [0.09]
*Model Fit Indices*						
*Adj. R*^*2*^	.05	.07	0.11	0.10	0.21	0.32
Δ*R*^*2*^		.02	0.04	−0.01	0.11	0.11
*F*	9.04^***^	7.34^***^	10.23^***^	6.68^***^	8.70^***^	13.42^***^
*n*	614	612	611	513	486	486
*AIC/n*	6.28	6.26	6.21	6.18	6.04	5.90
*BIC/n*	6.33	6.32	6.28	6.28	6.20	6.07

*AD* Alzheimer’s disease, *AIC* Akaike Information Criterion, *BMI* Body Mass Index, *CSF* cerebrospinal fluid, *FAQ* Functional Activities Questionnaire, *GDS* Geriatric Depression Scale, *GFAP* glial fibrillary acidic protein, *LDL* low-density lipoprotein, *MMSE* Mini-Mental State Examination, *NfL* neurofilament light, *PET* positron emission tomography, *SBP* Systolic Blood Pressure

^***^ *p* <.001

^**^ p <.01

^*^p <.05

### Latent Path Analyses (LPA) of modifiable risk factors

#### LPA of MRFs on global cognitive function (MMSE)

**Fig. 2:**
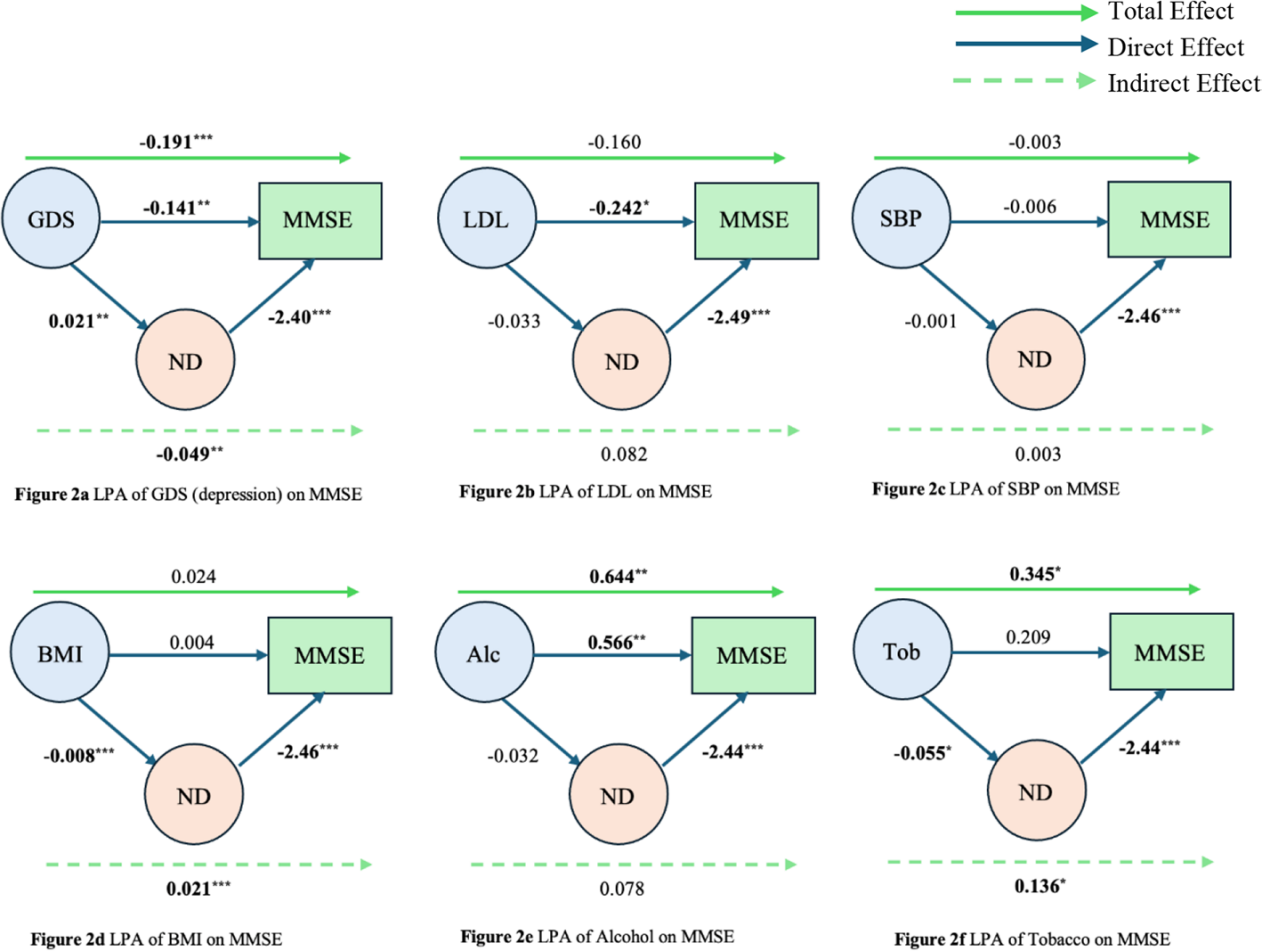
**a**-**f** Latent Path Analysis of Modifiable Risk Factors on Cognition measured by MMSE. *Note.* Unstandardised parameter estimates presented*. Bold* = significant; * p < .05, ** p < .01, *** p < *.001; Abbreviation.* Alc = Alcohol use; BMI = body mass index; GDS = Geriatric Depression Scale; LDL = Low-density lipoprotein cholesterol; LPA = Latent Path Analysis; MMSE = Mini-Mental State Examination; ND = plasma ADRD neurodegenerative disease pathology latent construct; SBP = Systolic Blood Pressure; Tob = Tobacco Use

Latent path analyses (LPAs) examining associations between MRFs, MMSE, and a latent construct of plasma ADRD neurodegenerative disease pathology (derived from five ADRD plasma biomarkers) are presented in Fig. 2a-f. All five plasma ADRD biomarkers consistently loaded significantly (loading factors > 0.4) on the latent construct across all LPAs conducted. In Fig. 2a, higher depressive symptoms (GDS scores) were significantly associated with greater ADRD neurodegenerative disease pathology (*B* = 0.021, *p* = 0.006), and lower MMSE scores (*B* = −0.141, *p* = 0.002). A statistically significant indirect association was observed consistent with pathway involvement (*B* = *-*0.049, *p* = 0.007) in relation to plasma ADRD neurodegenerative disease pathology, and accounted for 26% of the variance in MMSE. As shown in Fig. 2d, BMI had a modest inverse association with ADRD neurodegenerative disease pathology (*B* = −0.008, *p* < 0.001). The corresponding indirect association with MMSE was small, but positive (*B* = 0.021, *p* < 0.001), while the direct and total associations were non-significant (*p* > 0.05). Tobacco use showed a marginal, inverse association with ADRD neurodegenerative disease pathology (*B* = −0.055, *p* = 0.048; Fig. 2f). The direct path from tobacco use to MMSE was non-significant (*p* > 0.05). However, an indirect association involving ADRD neurodegenerative disease pathology reached statistical significance (*B* = 0.136, *p* = 0.049), and the total association with MMSE approached significance (*B* = 0.345, *p* = 0.051). The findings are consistent with a potential pathway in which lower plasma ADRD neurodegenerative pathology is associated with better cognitive performance in this sample.

The SRMR values across all LPA models were within acceptable range (0.059 to 0.063) [62]. However, the RMSEA values were elevated above the 0.08 threshold (GDS: 0.124; BMI: 0.129; Tobacco Use: 0.123). CFI values ranged from 0.828 to 0.839 and TLI values ranged from 0.732 to 0.750. Overall, the models showed convergence and plausibility, but the overall fit statistics indicate future refinement or the addition of other variables to improve the model structure. Thus, the borderline significant findings for tobacco use may reflect residual confounding not captured in the LPA model or model misspecification rather than a true protective mechanism and warrant cautious interpretation.

#### LPA of MRFs on Functional Impairment (FAQ)

**Fig. 3:**
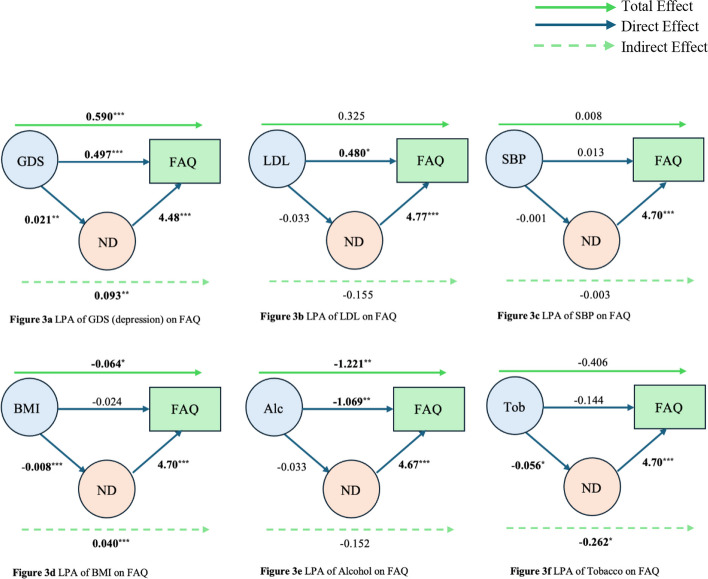
**a**-**f** Latent Path Analysis of MRFs on Functional Impairment measured by FAQ. *Note.* Unstandardised parameter estimates presented*. Bold* = significant; * p < .05, ** p < .01, *** p < *.001; Abbreviation.* Alc = Alcohol use; BMI = body mass index; FAQ = Functional Activities Questionnaire; GDS = Geriatric Depression Scale; LDL = Low-density lipoprotein cholesterol; LPA = Latent Path Analysis; ND = plasma ADRD neurodegenerative disease pathology latent construct; SBP = Systolic Blood Pressure; Tob = Tobacco Use

LPAs examining associations between MRFs, functional impairment (FAQ), and plasma ADRD neurodegenerative disease pathology are presented in Fig. 3a-f. Consistent with our MMSE findings, depressive symptoms, BMI, and tobacco use demonstrated statistically significant indirect associations on FAQ involving ADRD neurodegenerative disease pathology. As seen in Fig. 3a, higher depressive symptom severity (GDS) was associated with greater ADRD neurodegenerative disease pathology burden (*B* = 0.02, *p* = 0.006) and greater functional impairment (*B* = 0.50, *p* < 0.001). Plasma ADRD neurodegenerative disease pathology, in turn, independently predicted higher FAQ scores (*B* = 4.48, *p* < 0.001). An indirect association consistent with pathway involvement was observed (*B* = 0.09, *p* = 0.007) alongside a significant total association (*B* = 0.59, *p* < 0.001) of GDS scores on FAQ were found. In Fig. 3d, higher BMI was inversely associated with ADRD neurodegenerative disease pathology (*B* = −0.008, *p* < 0.001), and through this pathway, indirectly associated with lower FAQ scores (indirect effect *B* = −0.04, *p* < 0.001) and a small total association (*B* = −0.06, *p* = 0.020). Similarly, Fig. 3f shows tobacco use was marginally associated with lower ADRD neurodegenerative disease pathology burden (*B* = −0.06, *p* = 0.050*)*, with an indirect association with FAQ (*B* = −0.26, *p* = 0.050), while the total association was not statistically significant (*p* > 0.05), suggesting potential confounding or unmodeled influences.

Overall, the model fit was acceptable across LPAs with some limitations. SRMR values ranged from 0.059 to 0.063, though RMSEA values were slightly elevated (0.124 to 0.129), and CFI (0.821 to 0.839) and TLI values (0.723 to 0.750) reflected moderate fit. While the core mediation pathways emerged as robust, and model convergence was successful, the unexpected protective associations for BMI and tobacco use on plasma ADRD neurodegenerative disease pathology should be interpreted with considerable caution.

## Discussion

This study examined the added value of plasma ADRD biomarkers in characterising relationships between MRFs, cognitive performance, and functional impairment in a large, diverse cohort of 1002 adults from the Bio-Hermes-001 cohort. We used hierarchical regression models and LPA to assess: 1) the incremental contribution of MRFs, and plasma ADRD biomarkers on cognitive performance, daily function, and 2) whether plasma ADRD neurodegenerative disease pathology, indexed by five plasma biomarkers, was involved in pathways linking MRFs with cognitive or functional outcomes. Our findings address central recommendations from the *2024 Lancet Commission*, highlighting the importance of improved measurement and the potential relevance of targeting biomarker-detectable processes involved in the relationships between MRFs and dementia risk to help inform future prevention strategies.

### Main findings and mechanistic insights explaining global cognitive function (MMSE)

We found that younger age, higher education, and non-Hispanic white ethnicity were the strongest predictors of global cognitive performance, together accounting for 15% of the variance in MMSE scores. These associations remained statistically significant after conservative Bonferroni correction indicating the dominant role of sociodemographic factors in shaping global cognition. This finding is consistent with the theoretical concept of cognitive reserve [65], where education and lifelong learning are protective against clinical ADRD neurodegenerative pathology despite accumulating brain changes. Global cognitive performance is also linked to broader socioeconomic and sociocultural disparities, particularly due to racial and ethnic inequities in accessing (quality) education and healthcare which may influence cognitive aging trajectories in later life rather than intrinsic biological differences [66]. Lifestyle and mental health factors contributed minimally to explained variance in MMSE scores. Although depressive symptoms showed an inverse association with global cognition in partially adjusted models, this effect became insignificant after correction for multiple comparisons and was attenuated following inclusion of plasma ADRD biomarkers and functional impairment. This pattern may suggest that depressive symptoms reflect early manifestations of underlying neurodegenerative processes rather than being an independent driver of cognitive performance [67]. This is in line with prior work linking late-life depression to greater ADRD pathology burden and risk of cognitive decline [67, 68]. The nonsignificant findings between cardiometabolic and lifestyle factors and MMSE point to the possibility that metabolic influences on cognition occur as part of a more complicated or nonlinear mechanistic pathway not fully captured by our linear analyses. While BMI and smoking showed small protective effects and reduced neurodegeneration, these results likely reflect complex, bidirectional relationships not fully addressed here or survivor effects in older adults, reinforced by associations observed only in LPA, not regression analyses.

Among plasma ADRD biomarkers of neurodegenerative pathology, amyloid positivity emerged as the only significant biomarker that independently predicted poorer global cognitive function after Bonferroni correction in the fully adjusted regression model. This finding supports the central role of amyloid pathology in global cognitive decline, even in the presence of demographic and clinical heterogeneity. Higher plasma NfL and pTau217 were associated with lower MMSE scores in uncorrected analyses, but not in the fully adjusted model suggesting a smaller subset of plasma biomarkers are driving associations. Higher depressive symptoms initially predicted lower MMSE scores, but were not statistically robust after correction, suggesting that their contributions may be smaller, context-dependent, or mediated through functional impairment. Notably, when examined individually (Supplementary File 1), most plasma biomarkers (except A4β2/40) retained significant associations with MMSE scores under Bonferroni correction, highlighting shared variance when biomarkers are modelled simultaneously.

The inclusion of functional impairment (FAQ) produced the largest improvement in model fit, with higher FAQ scores strongly predicting lower MMSE global cognitive performance, even after correction. This suggests a close relationship between daily functional ability and global cognition, and supports that the MMSE may capture subtle cognitive changes before significant observable deficits of functional independence occurs across normal aging, mild cognitive impairment, and dementia [68]. Overall, in our final model, sociodemographic context and functional status appear to outweigh biological and lifestyle risk factors in explaining variance in global cognitive performance. Future research would benefit from integrating social determinants of health and functional assessments alongside plasma ADRD biomarkers when interpreting cognitive outcomes.

### Explaining Functional Impairment (FAQ)

Functional impairment was most strongly associated with depressive symptoms, elevated plasma NfL, and global cognitive performance (MMSE). Together, these variables explained 32% of the variance in total FAQ scores. Among these predictors, elevated NfL, a non-specific marker of neuronal injury and neurodegeneration in ADRD and Aβ positive patients with MCI [69], and lower MMSE scores emerged as the most reliable predictors of functional impairment after Bonferroni correction. LPA confirmed plasma ADRD neurodegeneration mediated the effects of depression on functional impairment. The mediation pathway connecting depression, plasma ADRD neurodegenerative disease pathology, and functional impairment supports the idea that depression influences functional outcomes indirectly, and co-occurs with, and may also accelerate, underlying disease processes rather than representing an isolated functional risk factor. As observed for cognitive outcomes, higher BMI and tobacco use were inversely associated with plasma ADRD neurodegenerative disease pathology burden and showed small protective associations with functional impairment. These effects were modest and should be interpreted cautiously, reflecting complex relationships.

### Key insights on depression and plasma ADRD neurodegenerative burden

Our findings lend support to the *2024 Lancet Commission* framework by identifying depression as a potential meaningful target for clinical intervention in preclinical or prodromal stages of ADRD given the influence on greater functional impairment and reduced cognitive performance via plasma ADRD biomarkers of neurodegenerative disease pathology. These findings align with existing evidence linking depression with elevated plasma ADRD biomarker burden, hastening amyloid plaque pathology and axonal damage, respectively [23, 67]. The present analyses did not directly assess biological mechanisms such as insulin resistance, oxidative stress, systemic inflammation, which are implicated with depression, and mechanistically linked with MRFs and cognitive decline [70–72]. Whether as antecedents to, resulting from, or exacerbated by depression, chronic inflammation and insulin resistance often co-occur with depression and disrupt brain glucose metabolism [70–73]. This is a consequence of impaired neuronal insulin signalling contributing to progressive neurodegenerative disease pathology and subsequent cognitive decline [21, 70]. Oxidative stress further contributes to overproduction of reactive oxygen species (ROS), highly reactive molecules that disrupt the balance between ROS and antioxidants causing neuronal damage and disease progression in both dementia and depression [74]. Given the relatively healthy and predominantly preclinical nature of the Bio-Hermes-001 cohort, late-life depression may represent a prodromal indicator of neurodegeneration, presenting a critical window for early identification and intervention to preserve mental health, cognitive function, and functional independence [73]. Multicomponent behavioural interventions (e.g., diet, exercise, cognitive-behavioural therapy) targeting mood, cardiometabolic health, and inflammatory risk factors simultaneously, especially in at-risk preclinical populations, may be particularly beneficial, and are now a WHO priority for primary and secondary dementia prevention [75, 76].

### BMI, physical activity, and cardiometabolic context

The observation that higher BMI had small neuroprotective effects on cognitive performance and functional impairment may reflect the so-called “obesity paradox,” where greater body mass is considered as a mid-life disease risk factor but may be protective in older adults providing metabolic reserves that buffer against neurodegenerative disease progression [77, 78]. These reserves may support metabolic regulation, neuroplasticity, and systemic resilience, helping maintain brain health and function [77]. Conversely, weight loss could be an early signal of preclinical neurodegenerative in changes in cognitive decline due to metabolic dysregulation or changes in appetite and physical functioning associated with dementia progression [79]. Survivor bias may have also played a role since this sample likely represents a healthier, more systemically resilient subgroup, which warrants cautious interpretation. Enrolled participants were considered cognitively normal or in the preclinical/early stages of disease, with late-stage dementia excluded.

There is emerging evidence that suggests physical activity (PA) may be associated with plasma ADRD neurodegenerative pathology. In a community cohort study of 739 cognitively healthy adults, higher self-reported PA levels were associated with significantly lower serum GFAP (β = −0.022), with effects modified by APOE ε4 carrier status [80]. However, exercise intervention studies have shown no consistent changes in circulating plasma biomarkers of pTau and NfL after 6-months of structured exercise [81]. Exploratory findings reported *higher* plasma GFAP and NfL in those with *higher* PA APOE ε4 non-carriers, with BMI mediating these associations [81]. Although PA was not a MRF assessed in the Bio-Hermes-001 cohort analysed in this study, our findings showing the neuroprotective role of higher BMI align with this emerging literature and suggest cardiometabolic health may play a central role in shaping biomarker profiles and downstream cognitive outcomes. Experimental studies showing an inverse association between BMI and plasma NfL, GFAP, and pTau suggest dilution effects related to blood volume and biological processes linked to aging and disease risk as factors [82], especially among APOE ε4 carriers, who tend to have accelerated BMI decline in later-life [82, 83]. BMI appears to represent a meaningful marker of preclinical disease processes and should be included in future longitudinal biomarker studies, alongside PA exposure and APOE ε4 status, across the transition from middle- to older adulthood. Clinical trials in late-life may also benefit from emphasising weight *maintenance* treatment approaches [84], instead of weight *loss* interventions*,* in certain subgroups to delay or slow cognitive decline in older adulthood.

### Alcohol and tobacco use: cautious interpretation

Alcohol consumption and tobacco use showed modest unexpected associations with better global cognition and functional outcomes. However, these findings must be interpreted with extreme caution and not as an endorsement for these behaviours. In the Bio-Hermes-001 cohort, alcohol and tobacco use were measured via a single self-report item and expressed as a positive or negative response to “current” or “past” use, without specification of the type, quantity or frequency of consumption, implicating the interpretability of non-linear or threshold effects. Sampling characteristics, survivor bias, measurement issues, or health behaviour inertia due to lack of perceived harm or absence of severe clinical illness [85] may further explain these patterns. Smokers or drinkers were most prevalent in the ‘cognitively normal’ strata, possibly representing a resilient subgroup. Additionally, individuals without a formal medical diagnosis of cognitive impairment may be less likely to modify lifestyle behaviours in the absence of a health diagnosis [86, 87] or may lack awareness or have ambivalence towards adopting healthier lifestyles for cognitive benefit [88]. Although a small body of research suggests a J-shaped curve between alcohol consumption and mortality or vascular health [89, 90] where moderate alcohol use may enhance vascular blood flow [91], reduce inflammation [90], and regulate HDL cholesterol [90, 92], the present data do not permit inference regarding beneficial effects. Additionally, given the lack of information in the Bio-Hermes-001 dataset on alcohol use, our analytic approach did not permit us to evaluate non-linear or J-shaped associations between alcohol use and cognitive or functional outcomes. Distinctions between current and former use, and potential differential effects related to duration, frequency, and type of alcohol (e.g., spirits, wine, beer) or tobacco (e.g., cigarettes, e-vapes, chewing tobacco) use would be helpful. Future longitudinal studies with detailed exposure assessments examining dose–response trajectories are needed to establish directionality of the mechanisms linking lifestyle-related risk factors and cognitive decline and functional impairment in late-life. Consistent with the 2024 *Lancet Commission,* MRFs in late life may show weaker or even reversed associations with cognitive and functional outcomes compared to midlife exposures. Thus, the timing of exposure across the transition from mid- to late-life remains a critical factor when interpreting MRFs, since associations in late-life potentially diverge from mid-life patterns.

### Limitations and future directions

Several limitations should be acknowledged. First, the cross-sectional design limits our ability to determine the direction of relationships or establish causality among MRFs, ADRD biomarkers, and cognitive and functional outcomes. Although LPA can estimate indirect associations, mediation cannot be inferred, and the observed pathways should be interpreted as statistical patterns rather than evidence of causal mechanisms. Second, for plasma biomarker data, precise measurements for pTau181 (*n* = 1) and pTau217 (*n* = 182) were missing due to the values falling below the lower limit or above the higher limit of assay quantitation. We followed the same imputation procedures for pTau217 and pTau181 outlined in the original cohort citation for imputing censored blood biomarker data [44]. Sensitivity analysis that excluded these imputed values was not conducted and we deemed this reasonable since exclusion would have left- or right-truncated the data and may have biased the estimates of the associations between blood biomarkers and outcomes. For other blood biomarkers (e.g., GFAP, NfL), blood pressure, and BMI measures, very small numbers (< 1% of the sample) were removed and deemed as outliers and unlikely to have affected the results significantly. We acknowledge that some data were missing for education level and other lifestyle factors consistent with prior large cohort studies (e.g., English Longitudinal Study of Ageing [ELSA]) [93]; we conducted complete-case analyses for these variables without imputation which may have affected results. Third, LPA model fit indices suggest potential structural limitations of the specified models warranting further investigation. These findings may reflect unmodeled heterogeneity, omitted pathways, or alternative latent structures not captured in our analytic framework. Future longitudinal data and alternative model specifications may help improve model fit and further clarify relationships. Fourth, key biomarkers, such as inflammatory markers (e.g., hs-C-reactive Protein, Interleukin-6), measures of insulin resistance and fasting blood glucose were not available in the dataset and limited a more comprehensive understanding of underlying biological mechanistic pathways. Fifth, we chose to group APOE ε4 with plasma biomarkers of neurodegeneration to ensure all variables derived from blood assays were considered together to reduce potential confounding related to differences in measurement type. Future research should consider including APOE ε4 as a covariate in demographic or risk factor models to further clarify its role in cognitive and functional outcomes.

We acknowledge that the MMSE was designed as a brief screening tool and proxy measure of global cognitive status, rather than a diagnostic or cognitive domain-specific tool for dementia. Although widely used in clinical and research settings, the MMSE has recognised psychometric limitations, including ceiling effects in highly educated participants, and potential bias related to language and education [94]. In our study, findings were based on MMSE total score and should be interpreted as reflecting global cognitive status, rather than definitive evidence of diagnostic classification or clinical impairment.

Several MRFs identified in the 2024 *Lancet Commission for Dementia Prevention, Intervention and Care * [19] could not be examined due to a lack of available data in the Bio-Hermes-001 cohort (e.g., dietary pattern, physical activity). This limited our ability to capture the full contribution of key MRFs of cognitive and functional outcomes and may have potentially resulted in residual confounding. Therefore, we emphasise that the present findings in our study be interpreted within the context of the available data. Future longitudinal studies incorporating more comprehensive assessments of MRFs would help fully characterise relationships.

Finally, although the Bio-Hermes-001 cohort is a uniquely diverse cohort (25% ethnic minority) and was sufficiently balanced across genders, it was not representative of the ethnic makeup of the country (USA) that participants resided [95], reflecting the broader issue of limited racial and ethnic diversity in dementia research.

Despite these limitations, our data support the integration of plasma ADRD neurodegenerative disease pathology biomarkers, particularly a smaller subset containing pTau217, NfL, and amyloid, for early cognitive assessment, stratification, and as treatment markers in clinical trials. Unlike prior work that has examined lifestyle factors or biomarker associations in isolation, our study extends existing literature by applying LPA to examine how plasma ADRD biomarkers are associated with relationships between MRFs and cognitive and functional outcomes, offering insight into potential biological pathways that may link these factors. Notably, we observed a neuroprotective role of BMI in later life within this latent biomarker framework. Screening for depression, physical activity, and metabolic blood biomarkers (e.g., insulin resistance, cholesterol) may be a potentially meaningful approach to improve early identification of adults most at-risk for neurodegeneration and cognitive decline. Biomarker-driven strategies may reduce dementia misclassification and strengthen mechanistic inference on MRFs. Additionally, adding plasma ADRD neurodegenerative pathology biomarkers into longitudinal cohort studies may enhance causal inference and guide prevention strategies that target pathophysiology, which aligns with National Institute on Aging and Alzheimer’s Association (NIA-AA) [96] strategic framework supporting a biomarker-based definition of ADRD. Longitudinal studies should consider detailed patterns of lifestyle behaviours (e.g., diet, exercise, sleep), alongside a comprehensive panel of blood-based biomarkers, including markers of inflammation and metabolic dysfunction, to clarify mechanistic associations and explain how clusters of risk factors may influence disease progression which aids in tailoring of interventions to individual risk profiles. In addition, increasing racial and ethnic diversity in research will address disparities in cognitive performance, functional impairment, and biomarkers of ADRD, making findings more applicable to broader populations. Overall, our findings highlight the relevance of plasma ADRD biomarkers to relationships between MRFs and cognitive and functional outcomes, and provide insights into a biologically informed framework for dementia prevention.

## Conclusion and next steps

Plasma ADRD biomarkers of neurodegenerative disease pathology improve the ability to detect and explain associations between MRFs and cognitive and functional outcomes. The present findings support a biomarker-driven, multifactorial prevention approach targeting BMI and depression to promote brain health in ageing populations. Future longitudinal research should incorporate broader plasma biomarkers and detailed lifestyle data to clarify causal pathways and determine how and when to best intervene. Adding assessments of plasma ADRD biomarkers of neurodegenerative pathology, such as NfL, in preclinical population and clinical trials may help refine treatment and provide earlier, more precise care for people at risk of dementia.

## Supplementary Information

Supplementary Material 1.

## Data Availability

Data was released by Bio-Hermes through the AD Workbench hosted by AD Data Initiative. Individual participant data is available through the same platform pending agreement from AD Data Initiative. Study protocol, data dictionary and R scripts for the variables and analyses used in this manuscript are available from the corresponding author upon reasonable request upon manuscript publication.

## Abbreviations

Aβ: Beta-Amyloid
AD: Alzheimer’s Disease
ADDI: Alzheimer’s Disease Data Initiative
ADRD: Alzheimer’s Disease and Related Dementias
AIC: Akaike Information Criterion
APOE4: Apolipoprotein E ε4 Gene
BIC: Bayesian Information Criterion
BMI: Body Mass Index
BP: Blood Pressure
CFI: Comparative fit index
CN: Cognitively Normal
CSF: Cerebrospinal Fluid
CRP: C-Reactive Protein
DBP: Diastolic Blood Pressure
FAQ: Functional Activities Questionnaire
GDS: Geriatric Depression Scale
GFAP: Glial Fibrillary Acidic Protein
HDL: High-Density Lipoprotein Cholesterol
IL-6: Interleukin-6
LDL: Low-Density Lipoprotein Cholesterol
LPA: Latent Path Analysis
MCI: Mild Cognitive Impairment
MMSE: Mini-Mental State Examination
MRF: Modifiable Risk Factor
ND: Plasma ADRD neurodegenerative disease pathology latent construct
NfL: Neurofilament Light
PAD: Probable Alzheimer’s Disease
PET: Positron Emission Tomography
pTau: Phosphorylated Tau
RMSEA: Root Mean Square Error of Approximation
ROS: Reactive Oxygen Species
SBP: Systolic Blood Pressure
SRMR: Standardised Root Mean Square Residual
TDP-43: TAR DNA-binding protein 43
TLI: Tucker and Lewis Index
TNF- α: Tumour Necrosis Factor-Alpha
tTau: Total Plasma Tau
