# Supplementary material for: Associations of plasma biomarkers of Alzheimer’s disease pathology with modifiable risk factors and cognitive and functional outcomes: evidence from cross-sectional prediction and latent path analyses in the Bio-Hermes-001 cohort
Source: Alzheimers Res Ther. 2026 Jun 18;18:180. doi: 10.1186/s13195-026-02106-4 (PMC13445916; doi:10.1186/s13195-026-02106-4)
Supplement: Supplementary file 1 [file 13195_2026_2106_MOESM1_ESM.docx]

**Supplementary File 1**

| **Table S1** Data imputation and outlier removal | | |
| --- | --- | --- |
| **Variable** | **Processing** | **# of observations removed** |
| pTau181 | Value marked as “<0.1” (n=1) set to 0 pg/mL | 0 |
| pTau217 | Based on procedures followed by Mohs et al.^1^, for values below the lower limit of quantitation (n=175), random values between 0 and 0.131 U/mL (minimum specified value) were assigned. For values above the upper limit of quantitation (n=7), the maximum specified value of 1.39 U/mL was assigned.  Mean = 0.247 U/mL, SD = 0.224. Hence 1.39 U/mL ceiling is around 5 SDs above mean value. | 0 |
| Aβ42 | Outliers defined as six standard deviations or more above the mean removed.  Mean = 53.9 pg/mL, SD = 54.0; 10 values between 437.6 and 733.7 pg/mL removed. | 10 |
| GFAP | Outliers defined as six standard deviations or more above the mean removed  Mean = 112.6 pg/mL, SD = 64.6; 2 values between 634.0 and 712.7 pg/mL removed. | 2 |
| NfL | Outliers defined as six standard deviations or more above the mean removed  Mean = 3.528 pg/mL, SD = 2.757; 4 values between 20.41 and 43.96 pg/mL removed. | 4 |
| Blood pressure | High blood pressure was indicated if systolic blood pressure (SBP) was above or equal to 140 mmHg or diastolic blood pressure (DBP) was above or equal to 90 mmHg^2^. Removed SBP values below 20 mmHg (n=4) as outliers or errors and DBP values above 800 mmHg (n=2). | 6 |
| Weight | Obese if body mass index (BMI) ≥30; overweight if 25≤ BMI <30. Removed BMI values above 4,000 (n=2) | 2 |
| Depression | Depression if the Geriatric Depression Scale (GDS, range 0-15) score ≥5^3,4^ or if participant has medical record of ongoing depression or major depression | 0 |
| **Abbreviation:** SD: standard deviation | | |

**Citations:**

1.	Mohs RC, Beauregard D, Dwyer J, et al. The Bio-Hermes Study: biomarker database developed to investigate blood-based and digital biomarkers in community-based, diverse populations clinically screened for Alzheimer’s disease. *Alzheimers Dement*. 2024;20(4):2752-2765.

2.	Flack JM, Adekola B. Blood pressure and the new ACC/AHA hypertension guidelines. *Trends Cardiovasc Med*. 2020;30(3):160-164.

3.	Sheikh JI, Yesavage JA. Geriatric Depression Scale (GDS): recent evidence and development of a shorter version. In: *Clinical Gerontology*. Routledge; 2014:165-173.

4.	Greenberg SA. The geriatric depression scale (GDS). *Best Pract Nurs Care Older Adults*. 2012;4(1):1-2.

| **Table S2** ADRD Biomarkers of Neurodegeneration, Cognition and Functional Outcomes by Mental Illness, Cardiometabolic, and Lifestyle Risk Factors (n = 1002) | | | | | | | | | |
| --- | --- | --- | --- | --- | --- | --- | --- | --- | --- |
| Modifiable Risk factor | | Amyloid Positivity in PET/CSF Yes/No, n/n, (% yes) | Aβ42/Aβ40^*^, mean (SD) | pTau181 (pg/mL), mean (SD) | pTau217 (U/mL), mean (SD) | NfL (pg/mL), mean (SD) | GFAP (pg/mL), mean (SD) | MMSE total, mean (SD) | FAQ total, mean (SD) |
| Education | ≥ 13 yrs | 44/69 (38.9) | 9.78 (1.04) | 1.12 (0.58) | 0.26 (0.25) | 3.70 (2.68) | 113.0 (62.3) | 25.3 (3.0) | 5.2 (6.4) |
|  | < 13 yrs | 157/316 (33.2) | 9.74 (0.96) | 1.04 (0.52) | 0.23 (0.20) | 3.39 (2.09) | 107.9 (58.3) | 26.9 (2.8) | 3.8 (5.5) |
|  | P-value | 0.296 | 0.684 | .121 | .105 | .179 | .408 | **<.0001** | **.020** |
| Depression^a^ | No | 303/554 (35.4) | 9.71 (0.99) | 1.07 (0.56) | 0.24 (0.22) | 3.35 (2.02) | 110.6 (57.7) | 26.7 (2.9) | 3.8 (5.5) |
|  | Yes | 42/52 (44.7) | 9.69 (0.97) | 1.20 (0.62) | 0.29 (0.25) | 4.02 (2.47) | 119.0 (73.2) | 25.9 (3.0) | 6.2 (7.3) |
|  | P-value | 0.095 | .843 | **.022** | .060 | **.002** | .176 | **.016** | **<.0001** |
| High LDL^b^ | No | 252/449 (35.9) | 9.67 (0.96) | 1.10 (0.59) | 0.25 (0.22) | 3.45 (2.05) | 113.4 (61.9) | 26.8 (2.8) | 3.8 (5.6) |
|  | Yes | 38/77 (33.0) | 9.83 (1.04) | 0.92 (0.43) | 0.19 (0.14) | 3.16 (1.84) | 106.3 (47.8) | 26.9 (3.0) | 3.6 (5.6) |
|  | P-value | .618 | .079 | **<.0001** | **.005** | .145 | .231 | .565 | .732 |
| Hypertension^c^ | No | 226/382 (37.2) | 9.683 (0.96) | 1.07 (0.56) | 0.25 (0.22) | 3.37 (1.96) | 110.7 (60.5) | 26.8 (2.8) | 3.6 (5.4) |
|  | Yes | 119/225 (34.6) | 9.75 (1.03) | 1.11 (0.60) | 0.24 (0.23) | 3.51 (2.29) | 112.6 (57.8) | 26.2 (3.1) | 4.7 (6.2) |
|  | P-value | .469 | 0.312 | .270 | .698 | .311 | .630 | **<.0001** | **.003** |
| Obesity^d^ | No | 267/402 (39.9) | 9.66 (0.99) | 1.11(0.57) | 0.26 (0.23) | 3.66 (2.14) | 120.0 (60.6) | 26.5 (3.0) | 4.2 (5.7) |
|  | Yes | 78/203 (27.8) | 9.81 (0.96) | 1.02 (0.56) | 0.21 (0.20) | 2.84 (1.84) | 90.5 (51.4) | 26.8 (2.7) | 3.6 (5.8) |
|  | P-value | **<.0001** | **.030** | **.022** | **<.0001** | **<.0001** | **<.0001** | .173 | .141 |
| Current/past use of alcohol | No | 101/174 (36.7) | 9.72 (0.97) | 1.11 (0.59) | 0.25 (0.24) | 3.57 (2.35) | 116.0 (64.6) | 26.0 (3.1) | 4.8 (6.3) |
|  | Yes | 244/433 (36.0) | 9.70 (0.99) | 1.07 (0.56) | 0.24 (0.22) | 3.36 (1.96) | 109.5 (57.2) | 26.9 (2.8) | 3.7 (5.5) |
|  | P-value | .900 | .757 | .339 | .550 | .155 | .116 | **<.0001** | **.005** |
| Current/past use of tobacco | No | 219/365 (37.5) | 9.69 (0.98) | 1.09 (0.59) | 0.25 (0.23) | 3.45 (2.17) | 113.0 (58.3) | 26.5 (3.0) | 4.1 (5.9) |
|  | Yes | 126/242 (34.2) | 9.74 (0.98) | 1.07 (0.53) | 0.23 (0.21) | 3.38 (1.94) | 108.9 (61.4) | 26.8 (2.7) | 3.9 (5.4) |
|  | P-value | .342 | 0.431 | .661 | .131 | .613 | .291 | .055 | .607 |
| **Abbreviation:** CSF: cerebrospinal fluid; Dep: Depression; FAQ: Functional Activities Questionnaire; GFAP: glial fibrillary acidic protein; HBP: High blood pressure; LDL: low-density lipoprotein; MMSE: Mini-Mental State Examination; NfL: neurofilament light; PET: positron emission tomography; SD: standard deviation.  ^a^ Depressed if GDS ≥5  ^b^ High if LDL ≥3.4 mmol/L  ^c^ High if DBP ≥90 mmHg or SBP ≥140 mmHg  ^d^ Obese if BMI ≥30  * Multiplied by 100 | | | | | | | | | |

**Table S2 Summary:** Depression (GDS ≥ 5) was associated with elevated circulating pTau181 (1.20 pg/mL vs. 1.07 pg/mL, *p* = .02) and NfL (4.02 pg/mL vs. 3.35 pg/mL, *p =* .002), and significant lower cognitive performance (MMSE 26.0 vs. 26.7, *p* = 0.02) and higher impairment scores (6.2 vs. 3.8, *p* <.0001) suggesting increased neurodegeneration and poorer cognitive function.

High LDL cholesterol was associated with lower pTau181 (0.92 pg/mL vs. 1.10 pg/mL, *p* <.0001) and pTau217 levels (0.19 vs. 0.25 U/mL, *p* = .005), and no differences observed in MMSE or FAQ scores. Participants with high blood pressure had significantly lower MMSE scores (26.2 vs. 26.8, *p* = .0001) and higher FAQ impairment scores (4.7 vs. 3.6, *p* = .003), suggesting impacts on cognitive performance and functional disability, but no significantly different plasma biomarker levels.

In contrast, a BMI ≥30 was associated with lower amyloid positivity (39.9% vs. 27.8%), and lower levels of pTau181 (*p* = .02), pTau217 (*p* < .0001), NfL (*p* <.0001), and GFAP (*p* <.0001). For lifestyle factors, current/past alcohol use was associated with higher MMSE score (26.9 vs. 25.9, *p* <.0001) and lower FAQ score (3.7 vs. 4.8, *p* =.006).

| **Table S3** ADRD biomarkers of neurodegeneration as independent predictors of MMSE Score | | | | | | |
| --- | --- | --- | --- | --- | --- | --- |
| Predictor | Model 5A | Model 5B | Model 5C | Model 5D | Model 5F | Model 5G |
| *Unstandardised Beta Coefficient (B*) [Standard Error] | | | | | | |
| Intercept | **27.94^***^**[2.48] | **30.01^***^**[1.84] | **30.70^***^**[1.83] | **28.92^***^**[1.89] | **28.81^***^**[1.90] | **29.90^***^**  [1.91] |
| **Demographics** | | | | | | |
| Age | **-0.09^***^**  [0.02] | **-0.07^***^**  [0.02] | **-0.09^***^**  [0.02] | **-0.07^***^**  [0.02] | **-0.06^**^**  [0.02] | **-0.08^***^**  [0.02] |
| Sex | 0.35  [0.25] | 0.31  [0.24] | **0.42^*^**  [0.24] | **0.49^*^**  [0.24] | **0.60^*^**  [0.25] | 0.39  [0.25] |
| Non-Hispanic White | **1.35^***^**  [0.29] | **1.45^***^**  [0.27] | **1.53^***^**  [0.27] | **1.46^***^**  [0.28] | **1.35^***^**  [0.28] | **1.35^***^**  [0.29] |
| Education | **0.17^***^**  [0.05] | **0.18^***^**  [0.04] | **0.17^***^**  [0.04] | **0.18^***^**  [0.04] | **0.19^***^**  [0.04] | **0.16^***^**  [0.04] |
| **Lifestyle** | | | | | | |
| BMI | 0.03  [0.02] | 0.02  [0.02] | 0.01  [0.02] | 0.01  [0.02] | 0.01  [0.02] | 0.02  [0.20] |
| Alcohol Use | **0.87^**^**  [0.27] | **0.87^**^**  [0.25] | **0.85^***^**  [0.25] | **0.75^**^**  [0.26] | **0.89^***^**  [0.26] | **0.81^**^**  [0.27] |
| Tobacco Use | 0.30  [0.24] | 0.25  [0.23] | 0.14  [0.23] | 0.22  [0.24] | 0.23  [0.24] | 0.26  [0.24] |
| **Mental Health** | | | | | | |
| Depression (GDS) | **-0.16^*^**  [0.07] | -0.09  [0.06] | -0.09  [0.06] | **-0.11^*^**  [0.06] | **-0.12^*^**  [0.06] | **-0.14^*^**  [0.07] |
| **Cardiovascular Health** | | | | | | |
| LDL | -0.20  [0.14] | **-0.28^*^**  [0.13] | **-0.28^*^**  [0.13] | -0.20  [0.13] | -0.15  [0.13] | -0.20  [0.14] |
| SBP | -0.01  [0.01] | -0.01  [0.01] | -0.01  [0.01] | -0.00  [0.01] | -0.01  [0.01] | -0.01  [0.01] |
| **ADRD Biomarkers of Neurodegeneration^a^** | | | | | | |
| Aβ42/40 | 0.19  [0.13] |  |  |  |  |  |
| pTau181 |  | **-1.68^***^**  [0.23] |  |  |  |  |
| pTau217 |  |  | **-0.05^***^**  [0.01] |  |  |  |
| NfL |  |  |  | **-0.29^***^**  [0.06] |  |  |
| GFAP |  |  |  |  | **-0.01^***^**  [0.00] |  |
| Amyloid Positivity in PET/CSF |  |  |  |  |  | **-1.63^***^**  [0.25] |
| *Model Fit* | | | | | | |
| *Adj. R^2^* | **0.15** | **0.24** | **0.25** | **0.19** | **0.17** | **0.21** |
| *F-statistic* | **9.46** | **15.34^***^** | **16.26^***^** | **11.9^***^** | **20.83^***^** | **12.72^***^** |
| *AIC/n* | 4.79 | 4.69 | 4.67 | 4.73 | 4.74 | 4.73 |
| **Note: *** *p <* .001*, ** p*** **< .01**, ****p* <.05;** AD: Alzheimer’s Disease; AIC: Akaike Information Criterion; CSF: cerebrospinal fluid; BMI: Body Mass Index; FAQ: Functional Activities Questionnaire; GDS: Geriatric Depression Scale; GFAP: glial fibrillary acidic protein; LDL: low-density lipoprotein; MMSE: Mini-Mental State Examination; NfL: neurofilament light; PET: positron emission tomography; SBP: Systolic Blood Pressure | | | | | | |

^a^Models 5A-5G represent separate prediction models with identical covariate structures. Each model includes one ADRD biomarker entered individually (not stepwise). Results are shown separately for interpretability, whereas primary models in the main manuscript include all biomarkers simultaneously.

| **Table S4** ADRD biomarkers of neurodegeneration as independent predictors of FAQ Score | | | | | | |
| --- | --- | --- | --- | --- | --- | --- |
| Predictor | Model 5A | Model 5B | Model 5C | Model 5D | Model 5F | Model 5G |
| *Unstandardised Beta Coefficient (B*) [Standard Error] | | | | | | |
| Intercept | 2.96  [4.95] | -4.99  [3.72] | -6.13  [3.74] | -2.25  [3.76] | -2.43  [3.80] | -2.56  [3.80] |
| **Demographics** | | | | | | |
| Age | **0.10^**^**  [0.04] | **0.07^*^**  [0.04] | **0.11^**^**  [0.04] | 0.06  [0.04] | 0.05  [0.04] | **0.08^*^**  [0.04] |
| Sex | **-1.14^*^**  [0.51] | **-1.15^*^**  [0.48] | **-1.34^**^**  [0.49] | **-1.49^**^**  [0.49] | **-1.72^***^**  [0.49] | **-1.41^**^**  [0.50] |
| Non-Hispanic White | -0.58  [0.58] | -0.65  [0.56] | -0.74  [0.56] | -0.72  [0.56] | -0.49  [0.56] | -0.76  [0.58] |
| Education | -0.09  [0.09] | -0.10  [0.09] | -0.08  [0.09] | -0.10  [0.09] | -0.12  [0.09] | -0.09  [0.09] |
| **Lifestyle** | | | | | | |
| BMI | -0.06  [0.04] | -0.04  [0.04] | -0.04  [0.04] | -0.03  [0.04] | -0.03  [0.04] | -0.05  [0.04] |
| Alcohol Use | **-1.42^**^**  [0.53] | **-1.43^**^**  [0.51] | **-1.40^**^**  [0.52] | **-1.19^*^**  [0.52] | **-1.48^**^**  [0.52] | **-1.43^**^**  [0.53] |
| Tobacco Use | -0.71  [0.49] | -0.65  [0.47] | -0.49  [0.47] | -0.57  [0.47] | -0.60  [0.48] | -0.75  [0.48] |
| **Mental Health** | | | | | | |
| Depression (GDS) | **0.56^***^**  [0.13] | **0.43^***^**  [0.13] | **0.46^***^**  [0.13] | **0.45^***^**  [0.13] | **0.50^***^**  [0.13] | **0.49^***^**  [0.13] |
| **Cardiovascular Health** | | | | | | |
| LDL | 0.33  [0.28] | 0.45  [0.27] | 0.43  [0.27] | 0.28  [0.27] | 0.21  [0.27] | 0.28  [0.27] |
| SBP | **0.03^*^**  [0.01] | 0.03  [0.01] | 0.03  [0.01] | 0.02  [0.01] | **0.03**  [0.01] | 0.02  [0.01] |
| **Plasma AD Biomarkers** | | | | | | |
| Aβ42/40 | **-0.69^**^**  [0.26] |  |  |  |  |  |
| pTau181 |  | **3.08^***^**  [0.45] |  |  |  |  |
| pTau217 |  |  | **0.07^***^**  [0.01] |  |  |  |
| NfL |  |  |  | **0.70^***^**  [0.11] |  |  |
| GFAP |  |  |  |  | **0.02^***^**  [0.00] |  |
| Amyloid Positivity in PET/CSF |  |  |  |  |  | **3.09^***^**  [0.50] |
| *Model Fit* | | | | | | |
| *Adj. R^2^* | **0.11** | **0.17** | **0.17** | **0.16** | **0.15** | **0.16** |
| *F-statistic* | **6.72^***^** | **10.79^***^** | **10.26^***^** | **9.94^***^** | **9.51^***^** | **9.38^***^** |
| *AIC/n* | 6.17 | 6.10 | 6.11 | 6.10 | 6.12 | 6.10 |
| **Note: *** *p <* .001*, ** p*** **< .01**, ****p* <.05;** AD: Alzheimer’s Disease; AIC: Akaike Information Criterion; BMI: Body Mass Index; CSF: cerebrospinal fluid; FAQ: Functional Activities Questionnaire; GDS: Geriatric Depression Scale; GFAP: glial fibrillary acidic protein; LDL: low-density lipoprotein; MMSE: Mini-Mental State Examination; NfL: neurofilament light; PET: positron emission tomography; SBP: Systolic Blood Pressure | | | | | | |

^a^Models 5A-5G represent separate prediction models with identical covariate structures. Each model includes one ADRD biomarker entered individually (not stepwise). Results are shown separately for interpretability, whereas primary models in the main manuscript include all biomarkers simultaneously.
